# Evaluation of the CCA Immuno-Chromatographic Test to Diagnose *Schistosoma mansoni* in Minas Gerais State, Brazil

**DOI:** 10.1371/journal.pntd.0004357

**Published:** 2016-01-11

**Authors:** Alda Maria Soares Silveira, Emanuele Gama Dutra Costa, Debalina Ray, Brian M. Suzuki, Michael H. Hsieh, Lucia Alves de Oliveira Fraga, Conor R. Caffrey

**Affiliations:** 1 Research Laboratory of Immunology, Faculty of the Health Sciences, Universidade Vale do Rio Doce (UNIVALE), Governador Valadares, Minas Gerais, Brazil; 2 Basic Department of Health Area, Universidade Federal de Juiz de Fora/Campus Governador Valadares (UFJF/GV),Governador Valadares, Minas Gerais, Brazil; 3 Center for Discovery and Innovation in Parasitic Diseases and the Department of Pathology, University of California San Francisco, San Francisco, California, United States of America; 4 Biomedical Research Institute, Rockville, Maryland, United States of America; 5 Children’s National Health System, Washington, District of Columbia, United States of America; 6 The George Washington University, Washington, District of Columbia, United States of America; Centers for Disease Control and Prevention, UNITED STATES

## Abstract

**Background:**

The Kato-Katz (KK) stool smear is the standard test for the diagnosis of *Schistosoma mansoni* infection, but suffers from low sensitivity when infections intensities are moderate to low. Thus, misdiagnosed individuals remain untreated and contribute to the disease transmission, thereby forestalling public health efforts to move from a modality of disease control to one of elimination. As an alternative, the urine-based diagnosis of schistosomiasis mansoni via the circulating cathodic antigen immuno-chromatographic test (CCA-ICT) has been extensively evaluated in Africa with the conclusion that it may replace the KK test in areas where prevalences are moderate or high.

**Methods and Findings:**

The objective was to measure the performance of the CCA-ICT in a sample study population composed of residents from non-endemic and endemic areas for schistosomiasis mansoni in two municipalities of Minas Gerais state, Brazil. Volunteers (130) were classified into three infection status groups based on duplicate Kato-Katz thick smears from one stool sample (2KK test): 41 negative individuals from non-endemic areas, 41 negative individuals from endemic areas and 48 infected individuals from endemic areas. Infection status was also determined by the CCA-ICT and infection exposure by antibody ELISA (enzyme-linked immunosorbent assay) to *S*. *mansoni* soluble egg antigen (SEA) and soluble (adult) worm antigen preparation (SWAP). Sensitivity and specificity were influenced by whether the trace score visually adjudicated in the CCA-ICT was characterized as positive or negative for *S*. *mansoni* infection. An analysis of a two-graph receiver operating characteristic was performed to change the cutoff point. When the trace score was interpreted as a positive rather than as a negative result, the specificity decreased from 97.6% to 78.0% whereas sensitivity increased from 68.7% to 85.4%. A significantly positive correlation between the CCA-ICT scores and egg counts was identified (r = 0.6252, *p* = 0.0001). However, the CCA-ICT misdiagnosed as negative 14.6% of 2KK positive individuals, predominantly those with light infections (fewer than 100 eggs/g feces). Considering 2KK as reference test, the discriminating power of the CCA-ICT (the area under the curve [AUC] = 0.817) was greater than the SEA-ELISA (AUC = 0.744) and SWAP-ELISA (AUC = 0.704).

**Conclusion:**

Our data for the performance of the CCA-ICT in the Brazilian communities endemic for schistosomiasis mansoni support those from Africa, *i*.*e*., in areas with greater infection prevalence and intensities, the CCA-ICT may be useful as a tool to indicate community-based preventative chemotherapy without individual diagnosis. However, because of the Brazilian Ministry of Health’s recommendation for individual diagnosis in areas where prevalence is less than 15%, *i*.*e*., those areas in which infection intensities are likely to be lowest, the CCA-ICT lacks the sensitivity to be used as standalone diagnostic tool.

## Author Summary

Detecting parasite eggs in stool by the Kato-Katz (KK) stool smear is the standard diagnostic test for infection with the flatworm parasite, *Schistosoma mansoni*. However, the test can miss those who have low burdens of infection, *i*.*e*., with few eggs in their feces. These misdiagnosed individuals, therefore, do not receive drug treatment and can continue to transmit the parasite into the environment putting the community at risk of infection. As an alternative diagnostic approach, the circulating cathodic antigen immuno-chromatographic test (CCA-ICT) is a simple-to-use handheld device (similar to a pregnancy test) that only needs urine to provide a quick and visual indication of whether one is infected or not. The consensus from studies in Africa is that the CCA-ICT could replace the KK test in those areas where people are more likely to harbor moderate to high worm burdens (*i*.*e*., more eggs in stool), but, like the KK test, it can miss those harboring light infection intensities. We evaluated the CCA-ICT performance in urine samples from 130 individuals living in areas non-endemic and endemic for schistosomiasis mansoni within the municipalities of Governador Valadares and Manhuaçu, Minas Gerais state, Brazil. The CCA-ICT performance characteristics, chiefly, sensitivity and specificity, depended on whether a ‘trace’ visual reading of the test was considered as a positive or negative diagnosis. We noted a positive correlation between the CCA-ICT scores and egg counts. However, the CCA-ICT misdiagnosed as negative about 15% of KK positive individuals, predominantly those with light infections. The CCA-ICT, nonetheless, had better discriminating power than commonly used antibody-based tests. We conclude that the CCA-ICT offers reasonable performance to diagnosis *S*. *mansoni* infection. However, in areas where infections intensities are light, the test lacks the sensitivity to be used as standalone diagnostic tool.

## Introduction

Schistosomiasis is a chronic and morbid global disease caused by the *Schistosoma* blood fluke that resides in the cardio-vascular system. Official estimates are that 200 million are infected [1,2] but it has been suggested that as many as 700 million people are afflicted, either due to active disease or as a consequence of irreversible organ damage even after drug treatment [3,4] The disease is transmitted by intermediate snail hosts that infest streams, irrigation systems, canals, reservoirs and ponds commonly used by communities as their source of water. Of the major species of schistosomes infecting humans, namely, *Schistosoma japonicum*, *Schistosoma haematobium* and *Schistosoma mansoni*, the latter is the solely responsible for disease in South America, including Brazil [5].

Treatment and control of schistosomiasis relies solely on the World Health Organization (WHO) recommended drug, praziquantel (PZQ) [1, 6–10]. PZQ is safe, reasonably effective, cheap to produce and administer, and quickly ameliorates morbidity [10–15]. Efforts to make PZQ more widely available through increased production and expanded treatment programs e.g. [6,16,17] are increasing, perhaps most notably demonstrated by the 2012 ‘London declaration’ whereby major pharmaceutical company, non-governmental organizations and philanthropic foundations pledged to make PZQ and other anthelmintic drugs more widely available (http://www.unitingtocombatntds.org/).

As PZQ administration increases, the incidence and prevalence of schistosomiasis are expected to decrease. Of concern, however, is that further progress to drive prevalence and incidence to zero is hindered by the insensitivity of the current ‘gold standard’, diagnostic Kato-Katz (KK) test to detect and facilitate treatment of intestinal schistosomiasis (caused by *S*. *mansoni* and *S*. *japonicum*) [18–20]. The KK test employs thick smears of feces (standardized to 41.7 mg on glass slide templates) and has a theoretical sensitivity of 24 eggs per gram (epg) of feces for a single slide. The test has worked effectively for 40 years in reducing global prevalence rates [21]. However, the principal and well known drawback of the KK test, particularly when employed according to the standard WHO format of two smears per stool [22] for community-based diagnosis and treatment, is its increasing insensitivity to diagnose what the WHO [22] stratifies as ‘moderate’ (100–399 epg) and ‘light’ (<100 epg) infection intensities [23–28], which will become more widespread due to expanded and more intense de-worming programs. Factors contributing to poor sensitivity include fecal stool consistency, and intra-specimen and day-to-day variation in fecal egg counts [23,29–33]. Consequently, infection prevalence is often seriously under-estimated due to missed diagnoses of infection [34, and references above]. Also, assessment of therapeutic efficacy post-treatment with PZQ can be confounded. Apart from the key issue of poor sensitivity, the KK test is a skill-intensive technique that requires time and trained personnel in the field, as well as microscopes and associated equipment [35]. Finally, and under-appreciated are the costs associated with the KK test, at least in the context of sub-Saharan Africa, which are between US$ 1.73 and US$ 6.89 [21,36–37]. Together, these detracting features of the KK test complicate any move from the modality of disease (morbidity) control to one of elimination. There is, therefore, a pressing need for a field-applicable, reliable and sensitive diagnostic tool.

Immunodiagnosis is generally more sensitive than examination of stool, particularly in low transmission areas in which infection intensities are light [6]. Typical antibody-detection assays utilize crude antigen extracts such as schistosome egg antigen (SEA) or soluble adult worm antigen preparation (SWAP). However, parasite-specific antibodies can remain for years after the infection has been cleared. As a result, such assays are unable to distinguish between current and previous infections. Also, antibody levels in serum do not necessarily correlate with infection intensity as determined by epg feces [38].

Over the last several years in sub-Saharan Africa, major efforts, not least the SCORE initiative funded via the Bill and Melinda Gates Foundation [39] and references therein, have focused on understanding the utility of a rapid immunochromatographic test (ICT) to diagnose schistosomiasis and perhaps replace the KK test. This ICT, which is commercially available (Rapid Medical Diagnostics, Pretoria, RSA; http://www.rapid-diagnostics.com/), measures a parasite-derived ‘circulating cathodic antigen (CCA)’ in the urine of patients infected with *S*. *mansoni*. The recent data (referenced below) suggest that the CCA-ICT performance is good enough to warrant its application as a standalone tool in surveying disease prevalence and risk, and, thus, facilitate the deployment of PZQ therapy at the community level.

Our report measures the performance of the CCA-ICT in a sample study population comprising residents from non-endemic and endemic areas for schistosomiasis mansoni in Minas Gerais state, Brazil. We compared the performance of the CCA-ICT with the SEA-ELISA and SWAP-ELISA. From the data arising, we interpret the potential utility of the CCA-ICT in the context of how chemotherapy of schistosomiasis is promoted in Brazil.

## Materials and Methods

### Ethical permission

Ethical clearance for this study was obtained from the Ethical Research Committee of Universidade Vale do Rio Doce-UNIVALE (PQ 019/08-11). District health, participants and parents/legal guardians were informed about the purpose and procedures of the study. Parent/legal guardians provided written informed consent for their children to participate. Participation was voluntary and individuals could withdraw at any time and without further obligation. All parasitological results were coded and treated confidentially. Any individual who was determined to be positive for schistosomiasis, soil-transmitted helminthiases or intestinal protozoal infections was treated with the appropriate dose of PZQ, albendazole or metronidazole, respectively, produced and distributed by the Brazilian Ministry of Health (Farmanguinhos, Oswaldo Cruz Foundation, Jacarepaguá, RJ, Brazil).

### Study design and subjects

**Fig 1:**
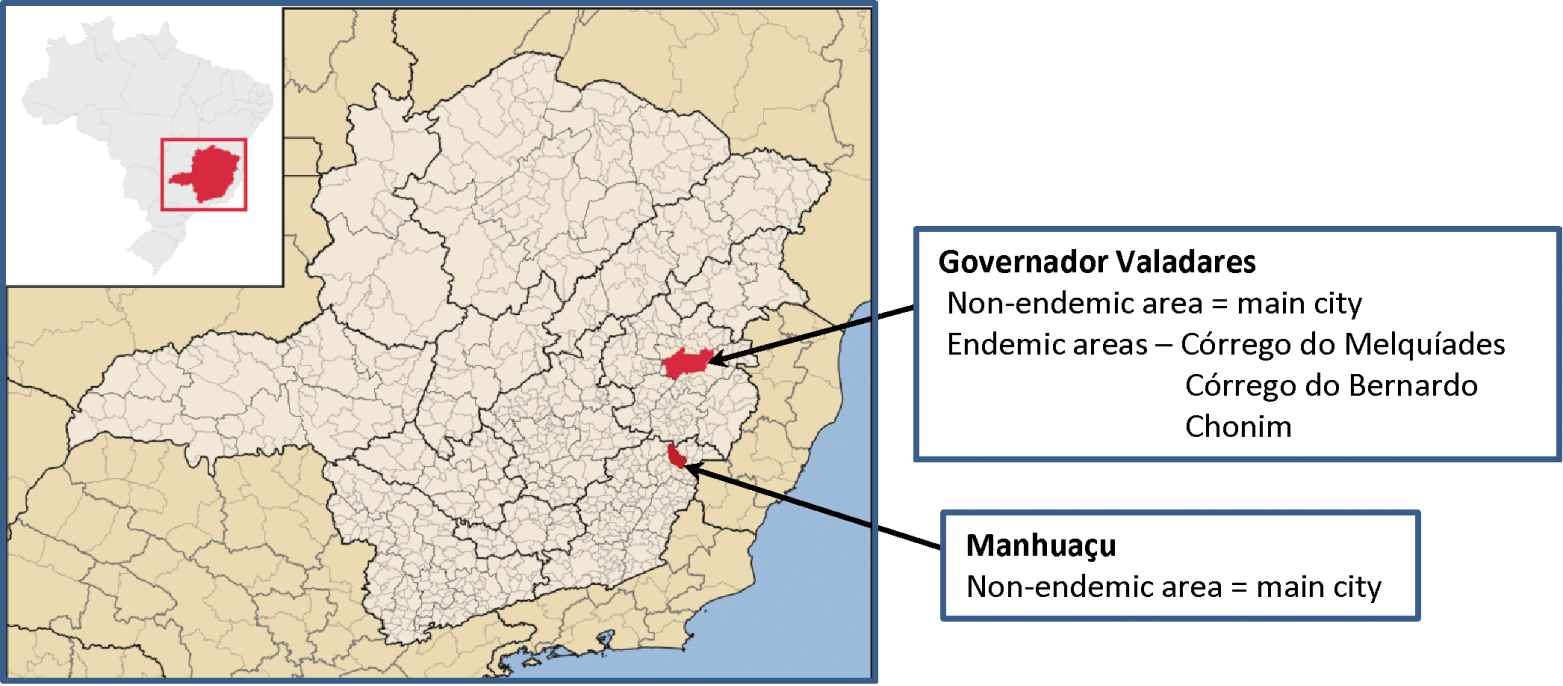
Map of Minas Gerais state in Brazil with the locations of the cities of Manhuaçu and Governador Valadares and associated rural villages, Córrego do Bernardo, Córrego do Melquíades and Chonim. (http://pt.wikipedia.org/wiki/Predefini%C3%A7%C3%A3o:Mapa_de_localiza%C3%A7%C3%A3o/Minas_Gerais)

The study was conducted from August 2012 to December 2013 in Governador Valadares and Manhuaçu, two municipalities in Minas Gerais state, Brazil (Fig 1). We enrolled 130 residents in non-endemic and endemic areas for schistosomiasis mansoni. The study design was based on a judgmental sampling using a non-probabilistic approach. Stool and urine samples were collected on the same day. As described below, parasitological stool examination by the KK [20] and Hoffmann-Pons-Janer (HPJ) tests [40] was performed immediately and the urine samples were frozen for subsequent analysis by CCA-ICT by a separate laboratory team that was blinded to the results of the KK/HPJ tests. Upon data mining, the KK results were categorized into three study groups; individuals resident in non-endemic areas who were negative on 2 KK slides (“2KK-NEG non-endemic area”; n = 41), individuals resident in endemic areas who were negative on 2 KK slides (“2KK-NEG endemic area”; n = 41) and individuals resident in endemic areas who were positive for *S*. *mansoni* eggs on at least 1 of 2 slides (“2KK-POS endemic area”; n = 48). The 2KK-NEG endemic area individuals had a previous history of at least three negative stool tests for *S*. *mansoni* despite being exposed to sources of infection. The 2KK-NEG non-endemic subjects had no previous history of *S*. *mansoni* infection, and neither received treatment for schistosomiasis nor had contact with sources of infection. Detailed information for the study groups is presented in Table 1.

**Table 1:** Characterization of the study groups.

	2KK-NEG	2KK-NEG	2KK-POS
	non-endemic area n = 41	endemic area n = 41	endemic area n = 48
**Age** (year)*	39.8 ± 19.1	51.7 ± 18.4	28.8 ± 19.1
**Range** (year)	10–93	17–89	7–80
**Gender**			
Male	10 (24%)	16 (39%)	29 (60%)
Female	31 (76%)	25 (61%)	19 (40%)
**2KK** (epg)**	0	0	276.0 ± 440.0
**Helminths**	0	1 (2.4%)	9 (18.8%)
*Hookworm*	0	0	7
*Enterobius vermicularis*	0	1	2
**Intestinal protozoa**	7 (17.1%)	22 (53.7%)	23 (47.9%)
*Entamoeba coli/histolytica*	5	22	19
*Giardia lamblia*	1	0	2
*E*. *coli/histolytica + G*. *lamblia*	1	0	2

*Arithmetic mean ± SD; 2KK, infection intensity calculated by reading two slides stool by the Kato-Katz method; epg, eggs per gram of feces

** arithmetic mean ± SD.

### Parasitological diagnosis

Diagnosis of *S*. *mansoni* was performed using the 2KK test (41.7 mg of stool per smear) [20]. The intensity of infection was expressed as eggs per gram (epg) of feces using the arithmetic mean of the egg count obtained from the two slides multiplied by 24. Infection intensity [22,41] was categorized as negative (0 epg), light (1–99 epg), moderate (100–399 epg) or heavy (≥ 400 epg). Thick smears were also examined for eggs of soil-transmitted helminths (hookworm, *Ascaris lumbricoides* and *Trichuris trichiura)* and *Enterobius vermicularis*. Each stool sample was also assessed by the HPJ sedimentation method for helminth eggs and protozoa (*Entamoeba* sp. and *Giardia lamblia)* [40]. The examinations were performed by expert technicians working at the Research Laboratory of Immunology, Universidade Vale do Rio Doce (UNIVALE). The data regarding intestinal protozoa infection was considered for sub-grouping the study population as Protozoa–and Protozoa + to evaluate the CCA-ICT performance (Table 2). Considering the very low rate of *S*. *mansoni* co-infection with other helminths (only nine out of 130 subjects), the effect of other helminth infections on CCA-ICT performance was not studied further.

**Table 2:** The results of the CCA-ICT* are not affected by infection with intestinal protozoa.

		CCA-ICT ^+^	CCA-ICT ^-^
2KK NEG (non-endemic area)^*a*^			
	All	9/41 (22.0%)	32/41 (78.0%)
	Protozoa +	1/7 (14.3%)	6/7 (85.7%)
	Protozoa -	8/34 (23.5%)	26/34 (76.5%)
2KK NEG (endemic area)^*a*^			
	All	5/41 (12.2%)	36/41 (87.8%)
	Protozoa +	4/22 (18.2%)	18/22 (81.8%)
	Protozoa -	1/19 (5.3%)	18/19 (94.7%)

*Cutoff = ‘trace’; CCA-ICT^+^ = CCA-ICT positive; CCA-ICT^-^ = CCA-ICT negative. Levels of significance were based on χ^2^ (categorical variables):

^*a*^*p* = 0.3499

^*b*^*p* = 1.000

### The CCA-ICT

A single urine sample from each individual was collected to test for the presence of *S*. *mansoni* CCA using the CCA-ICT. Urine samples were initially stored at -20°C then prepared as aliquots of 1 ml and frozen at -80°C before use. The CCA-ICT was performed according to the manufacturer’s instructions. Briefly, one drop of urine was added to the sample well of the ICT cassette and allowed to absorb. Then, one drop of buffer (provided with the kit) was added. The test score was read 20 min after adding the buffer. Positive color reactions were scored as trace (very light band), weak (+), medium (++) and strong (+++).

### *S*. *mansoni* adult worm soluble antigen (SWAP)

Female Swiss Webster mice (4–6 weeks old) were infected subcutaneously with 100 *S*. *mansoni* cercariae of the LE strain. After 45 days, the animals were sacrificed by cervical dislocation and perfused via the hepatic portal system using a 0.85% saline solution containing 50U/l heparin [42]. Adult worms were washed three times with 0.15 M phosphate-buffered saline, pH 7.2, mechanically grinding (Virtiz Precisa, Dietikon, Switzerland) and centrifuged at 9,500 *g* for 1 h at 4°C (Eppendorf AG, Hamburg, Germany). The supernatant was dialyzed using a cellulose membrane (Sigma-Aldrich D9777, St Louis, USA) against 0.9% saline for 48 h at 4°C. The material was then centrifuged at 1,250 *g* for 15 min at 4°C and the supernatant stored at -20°C. An aliquot was measured for protein content (Nanodrop, Thermo Scientific 2000, USA) in order to normalize protein concentration in the SWAP-ELISA used to detect human antibodies (see below).

### *S*. *mansoni* eggs soluble antigen (SEA)

After perfusion of infected mice, livers were removed to recover parasite eggs. The eggs were homogenized and mechanically ground (Virtiz Precisa) for 40 min in 0.85% saline. The homogenate was centrifuged at 9,500 *g* for 1 h at 4°C. After 48 h of dialysis with a cellulose membrane (Sigma-Aldrich) against a 0.9% saline solution, an aliquot of the supernatant was measured for protein content (Nanodrop) to normalize protein concentration in the SEA-ELISA used to detect human anti-egg antibodies (see below).

### SEA and SWAP-ELISA assays

ELISA tests were performed according to [43]. Microtiter plates (MaxiSorpTM Surface; NUNC, Denmark) were sensitized with 100 μl/well of antigen solution diluted in 0.05 M carbonate-bicarbonate, pH 9.6, for 16 h at 4°C. The plates were washed three times with washing buffer (0.15 M PBS, pH 7.2, 0.05% Tween 20 (LGC Biotecnologia, BR). Non-specific sites were blocked with 10% fetal bovine serum in washing buffer at 37°C for 1 h. After washing three times, 100 μl of serological sample diluted in PBS were added and the plates incubated at room temperature for 1 h. After washing three times, plates were incubated at room temperature for 1 h with conjugated anti-IgG human peroxidase (Sigma-Aldrich A0170 Lot: 062m4819, St. Louis, USA) diluted in washing buffer. After washing three times, 100 μl of substrate 3’,3’,5,5-tetramethylbenzidine (TMB/H_2_O_2_ Invitrogen T0440, St Louis, USA) were added to each well. The reaction was stopped after 20 min of incubation in the dark by addition of 50 μl/well of 2N sulfuric acid. Absorbance was measured at 450 nm in a microplate reader (Model 3550, Bio-Rad Laboratories,Tokyo, Japan). Each serum sample was used in duplicate in two separate assays. Average levels of optical density (OD) were determined from the quadruplicate measurements.

For the SWAP-ELISA, 1 μg/ml of SWAP was employed. Serum samples were diluted 1:50 and the conjugated secondary antibody was diluted 1:60,000. For the SEA-ELISA, 3 μg/ml of SEA was employed. Serum samples were diluted 1:150 and the conjugated secondary antibody was diluted 1:40,000.

### Statistical analysis

We used a judgmental sampling and a non-probability selection approach. Data were analyzed using GraphPad Prism software 5:03, STATA version 13 (College Station, Texas, USA) and SPSS, software version 11.5. Differences were considered significant when α was 0.05. The relationship between the intensity of infection determined by the 2KK test and the CCA-ICT results was examined by the Spearman correlation test. The chi-square test was used to analyze categorical variables. The power of association (the degree of agreement) between the 2KK and CCA-ICT was evaluated using Kappa (k) statistics where k <0 indicates an absence of agreement; k = 0 to 0.2 is poor concordance; k = 0.21 to 0.4 is weak agreement; k = 0.41 to 0.6 is moderate agreement, k = 0.61 to 0.8 is good agreement and k = 0.81 to 1.0 means excellent agreement [44,45]. The performance criteria of the CCA-ICT were sensitivity and specificity, positive and negative predictive values, positive and negative values of likelihood (LR) and the area under the curve (AUC). Cutoff estimates using the technical TG-ROC (Two-Graph Receiver Operating Characteristic) [46] and the ROC [47] were developed using the MedCalc, version 7.7.0.0 (Alexandria, USA). The AUC indicates the probability of accurately identifying true positives, where one could distinguish between non-informative (AUC = 0.5), less accurate (0.5<AUC≤0.7), moderately accurate (0.7<AUC≤0.9), highly accurate (0.9<AUC<1) and perfect tests (AUC = 1) [48]. Data from the 2KK test were used as the primary reference standard for diagnostic comparisons, and data from a combination of the 2KK test, SWAP-ELISA and SEA-ELISA served as the alternative reference test.

## Results

### ROC curve analysis to establish the CCA-ICT cutoff

**Fig 2:**
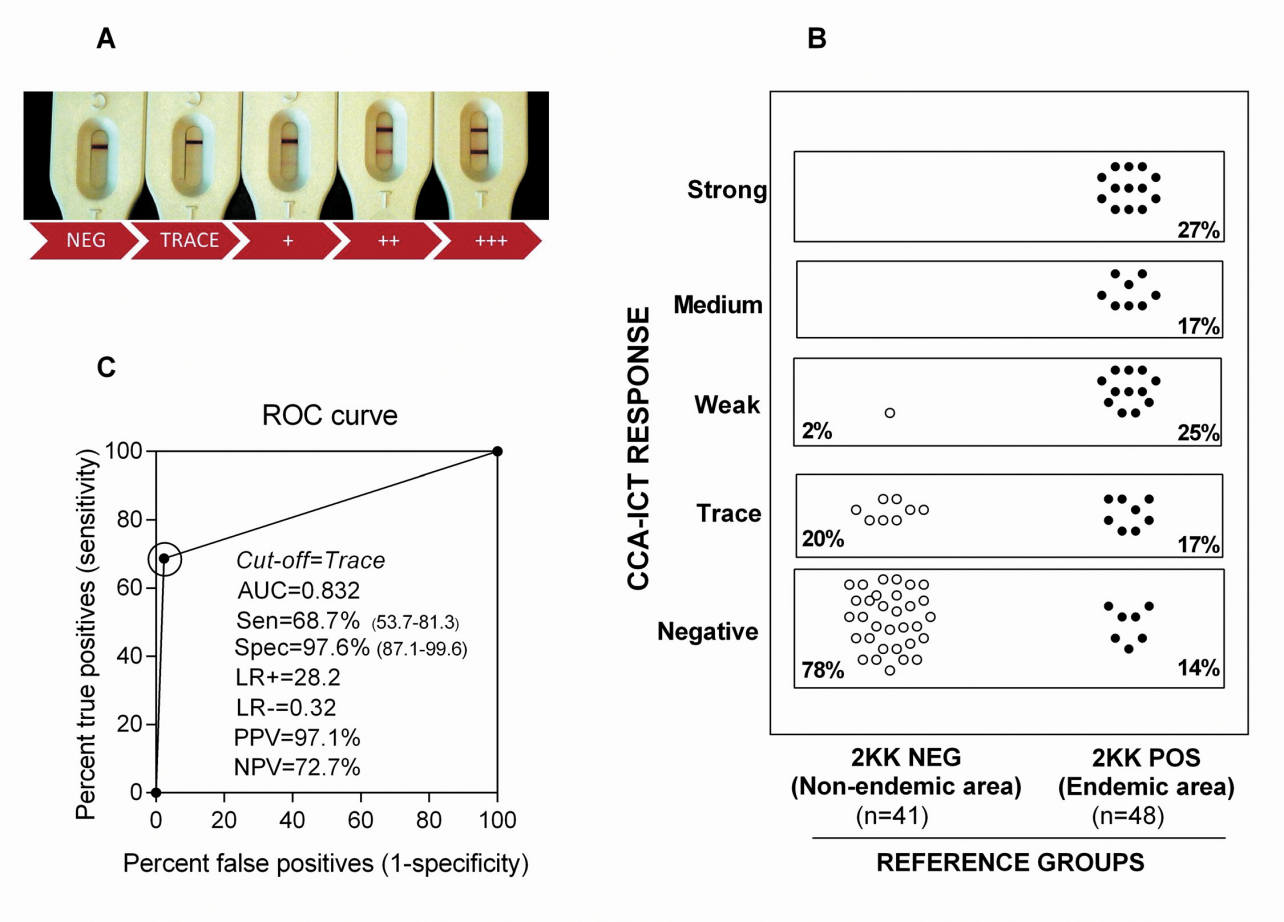
Performance of the CCA-ICT for diagnosing *S*. *mansoni* infection and receiver operating characteristic (ROC) curve analysis using two Kato-Katz thick smears (2KK test) as the reference. **A**: Photograph of the different reactions possible with the CCA-ICT: negative, trace, weak (+), moderate (++) and strong (+++). **B**: Distribution of the CCA-ICT results for “2KK-NEG non-endemic area” (n = 41) and “2KK POS endemic area” (n = 48). **C**: The ROC curve and the area under the curve (AUC) for the performance of the CCA-ICT. The ROC curve established the optimal cutoff point as the trace score. Likelihood ratio positive (LR+); likelihood ratio negative (LR-); Positive Predictive Value (PPV); Negative Predictive Value (NPV).

The CCA-ICT results were first categorized based on the adjudicated visual scores as represented in Fig 2A, ranging from negative through trace, weak (+) and medium (++) to strong (+++). Of the “2KK-NEG non-endemic area” samples, 78% were negative by the CCA-ICT whereas 22% were either trace or weakly positive. For the “2KK-POS endemic area” samples, 14% were negative by the CCA-ICT, and 17% (8/48), 25% (12/48), 17% (8/48) and 27% (13/48) were adjudicated as trace, weak, medium and strong, respectively (Fig 2B).

ROC curve analysis indicated that the trace score should be considered negative in order to segregate the “2KK-NEG non-endemic area” from the “2KK-POS endemic area” (Fig 2C). Sensitivity (SS) and specificity (SP) were 68.7% (95% confidence interval (CI): 53.7%-81.3%) and 97.6% (CI: 87.1%-99.6%), respectively, and the positive (PPV) and negative predictive values (NPV) were 97.1% (CI: 84.7%-99.9%) and 72.7% (CI: 59%-83.9%), respectively. The value for the area under the ROC curve (AUC), indicating how likely the CCA-ICT will make a correct diagnosis, was 0.832 (CI: 0.761–0.902; Fig 2C).

### CCA-ICT performance with ‘trace’ considered negative and correlation with the intensity of infection

**Fig 3:**
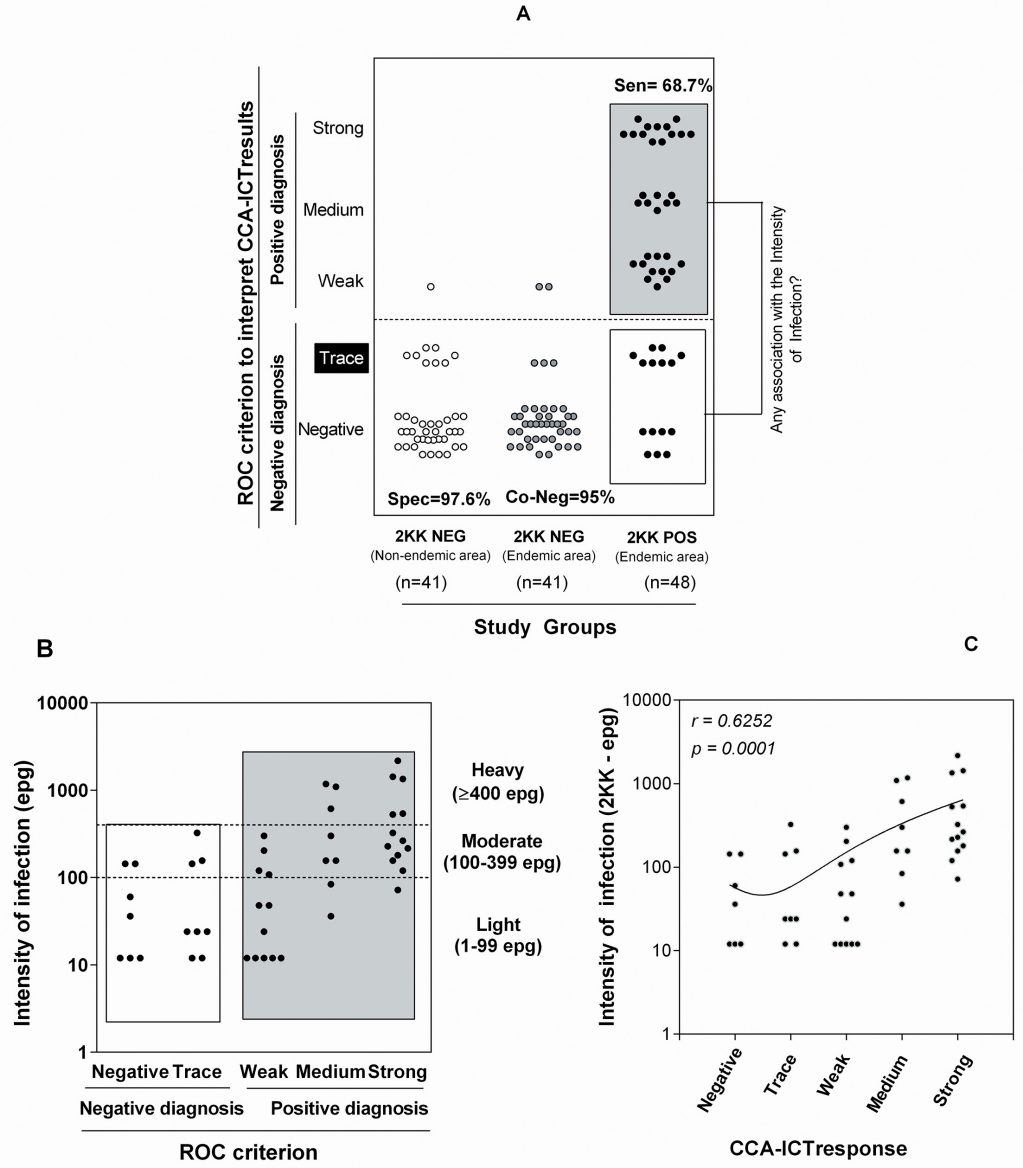
Performance comparisons between the 2KK test and the CCA-ICT with the cutoff set to trace. A: Distribution of the CCA-ICT scores of “2KK-NEG non-endemic area” (n = 41), “2KK NEG endemic area” (n = 41) and “2KK POS endemic area” (n = 48) when the trace CCA-ICT score was considered negative. B: Association between the CCA-ICT scores, the individual mean egg per gram (epg) values (plotted on a logarithmic scale) and classes of intensity of infection. According to the 2KK test, the percentage for each class of infection intensity was 43.8% light, 39.6% moderate and 16.6% heavy. C: Correlation between epg values and CCA-ICT scores showing a positive association with the CCA-ICT scores.

Fig 3A shows the distributions of the CCA-ICT results when ‘trace’ was considered a negative result. The performance indices demonstrated that the CCA-ICT showed a similar positivity for the “2KK-NEG non-endemic area” and the “2KK-NEG endemic area” (2.4% and 4.8%, respectively) with specificities of 97.6% and 95%, respectively. For the “2KK-POS endemic area”, the CCA-ICT falsely categorized 31% as uninfected (15/48: 7 negative and 8 trace responses). Fig 3B shows the association between the CCA-ICT scores for “2KK-POS endemic area” and the infection intensity classified by mean egg counts (epg) according to the WHO [21]. Interestingly amongst the “2KK-POS endemic area” persons displaying negative results in the CCA-ICT, 67% (10/15) and 33% (5/15) harbored light and moderate infections, respectively. For those “2KK-POS endemic area” with positive CCA-ICT, 33.3% (11/33), 42.4% (14/33) and 24.2% (8/33) harbored light, moderate and heavy infections, respectively. A significantly positive correlation between the CCA-ICT data and intensity of infection was identified (r = 0.6252, *p* = 0.0001; Fig 3C).The strength of association between CCA-ICT (positive and negative data) and 2KK test was also evaluated by the Kappa coefficient. A value of 0.663 (*p*<0.0001) was determined indicating a satisfactory agreement between the tests.

### CCA-ICT performance with ‘trace’ considered positive and effects of protozoa intestinal infections

**Fig 4:**
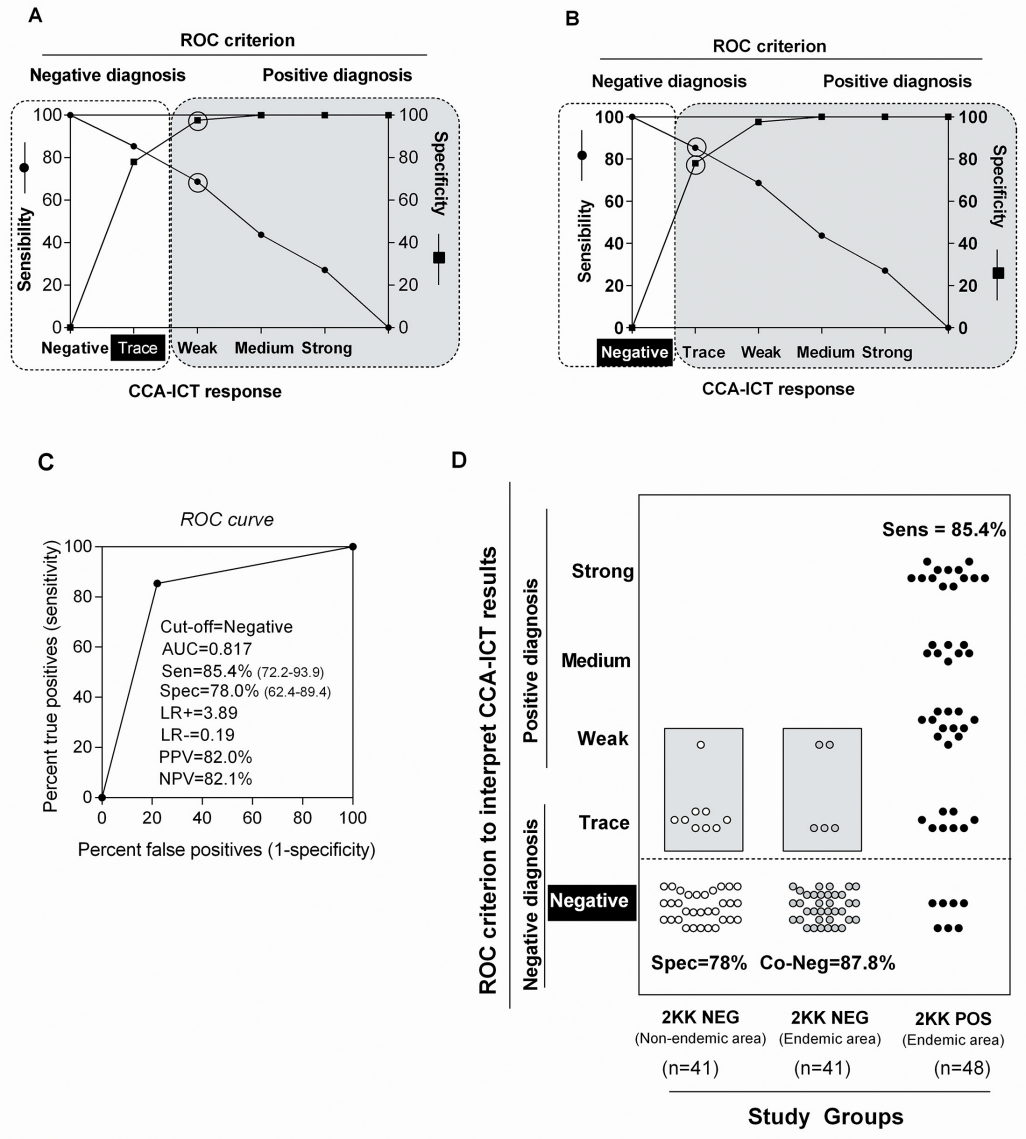
CCA-ICT performance with the cutoff set to negative. A: Two-graph receiver operating characteristic (TG-ROC) curve showing the cutoff for the CCA-ICT set to the trace score. B: as for A but with the cutoff set to the negative score. C: ROC curve and the AUC for the performance of CCA-ICT with the cutoff set to the negative score using “2KK-NEG non-endemic area” (n = 41) and “2KK POS endemic area” (n = 48) as the reference groups. D: Distribution of the CCA-ICT scores of “2KK-NEG non-endemic area” (n = 41), “2KK NEG endemic area” (n = 41) and “2KK POS endemic area” (n = 48) when the cutoff was set to the negative CCA-ICT score. The area under the curve (AUC); Likelihood ratio positive (LR+); Likelihood ratio negative (LR-); Positive Predictive Value (PPV); Negative Predictive Value (NPV)

As demonstrated above, 31% of “2KK-POS endemic area” individuals were misclassified as uninfected (false negatives) by the CCA-ICT when ‘trace’ was considered a negative result. Two-graph receiver operating characteristic (TG-ROC) analysis was, therefore, performed to recalculate the cutoff in order to enhance sensitivity. An advantage of the TG-ROC curve approach over the ROC curve is the more direct reading of the value of a cutoff point associated with a specific combination of sensitivity and specificity. The changes in the CCA-ICT performance when the TG-ROC curve cutoff was shifted from ‘trace’ considered as negative to 'trace' considered as positive are shown in Fig 4A and 4B, respectively. Sensitivity increased from 68.7% to 85.4% (CI: 72.2%-93.9%) but specificity dropped from 97.6% to 78% (CI: 62.4%-89.4%). The AUC maintained a moderate power of discrimination (0.817; CI: 0.736–0.899) (Fig 4C), whereas PPV and NPV were revised downwards and upwards, respectively (compare Figs 4C and 2C).

Shifting the TG-ROC curve cutoff from ‘trace’ to ‘negative’ resulted in fewer false negatives (7 or 14.6%; Fig 4D). It was also observed that among the seven misdiagnoses by the CCA-ICT in the “2KK-POS endemic area”, five had light infections and two had moderate infections (See Fig 3B). Interestingly, the cutoff shift led to an increase in false positives among the “2KK-NEG non-endemic area” (9/41) and “2KK-NEG endemic area” (5/41). This result prompted us to investigate a possible association with intestinal protozoan parasites. We compared the CCA-ICT scores from people infected with intestinal protozoa with individuals who were negative. The percentage of CCA-ICT positive results was not significantly different based on intestinal protozoa status, neither from “2KK-NEG endemic area” (*p* = 0.3499) nor “2KK-NEG non-endemic areas” (*P* = 0.1000), suggesting that intestinal protozoa infections do not influence the urine CCA-ICT results (Table 2).

### Association between CCA-ICT, SEA-ELISA and SWAP-ELISA

**Fig 5:**
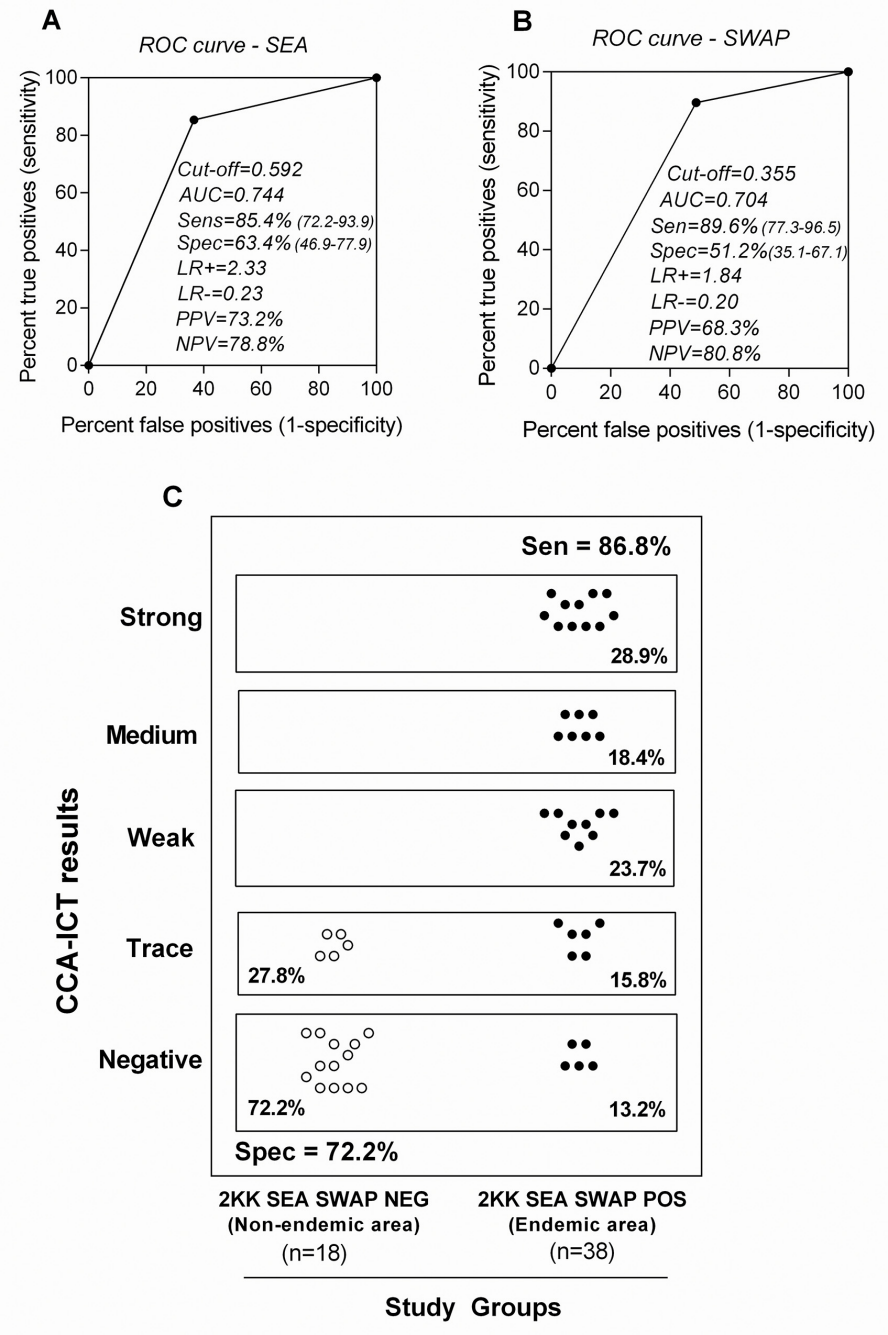
Association between the CCA-ICT, SEA- ELISA and SWAP-ELISA data. The ROC curves and AUC for the SEA-ELISA and SWAP-ELISA ELISA constructed for “2KK-NEG non-endemic area” (n = 41) and “2KK POS endemic area” (n = 48) are presented in **A** and **B**, respectively. The 2KK test was employed as the reference. **C:** Distribution of the CCA-ICT scores among those individuals categorized as “2KK SEA SWAP NEG” (n = 18) and “2KK SEA SWAP POS” (n = 38) when the trace CCA-ICT score was considered positive. The area under the curve (AUC); Likelihood ratio positive (LR+); Likelihood ratio negative (LR-); Positive Predictive Value (PPV); Negative Predictive Value (NPV).

The association between the CCA-ICT with serum antibody reactivity detected by SEA-ELISA and SWAP-ELISA is presented in Fig 5. At first, the ROC curve analysis for SEA-ELISA (Fig 5A) and SWAP-ELISA (Fig 5B) was carried out to estimate the cutoff and performance indices (sensitivity, specificity, PPV, NPV and AUC) using the “2KK-NEG non-endemic area” and “2KK-POS endemic area” as reference groups. The cutoffs of OD = 0.592 for the SEA-ELISA and OD = 0.355 for the SWAP-ELISA along with the respective AUC values of 0.744 (CI: 0.654–0.834) and 0.704 (CI: 0.615–0.793) were obtained. These AUC values were lower that obtained with the CCA-ICT (AUC = 0.817), when ‘trace’ was considered positive (Fig 4C). Upon analyses of the SEA-ELISA and SWAP-ELISA results, the study groups were redefined and referred to as “2KK SEA SWAP NEG” (n = 18) and “2KK SEA SWAP POS” (n = 38). For these analyses, the ‘trace’ was considered positive. Analysis of the data (Fig 5C) demonstrated that in 2KK SEA SWAP NEG, 27.8% (5/18) of subjects were classified as CCA-ICT positive, a proportion similar to the 22% obtained for the “2KK-NEG non-endemic area” (Fig 4D). Moreover, the CCA-ICT diagnosed as negative 13.2% (5/38) of subjects in the 2KK SEA SWAP POS, a proportion of false negatives similar to the 14.6% previously obtained for 2KK-POS endemic area (Fig 4D).

## Discussion

The expansion and acceleration of schistosomiasis control programs that rely on PZQ have successfully decreased the prevalence and the intensity of infections [41,49]. However, further gains are threatened by the low sensitivity of the current ‘gold-standard’ KK method [50–52], especially in light of findings that recurrent reinfection with low intensity infections can still lead to persistent morbidity [41,53–54]. Accurate and sensitive case detection is, therefore, paramount to controlling and ultimately eliminating schistosomiasis. Additional critical factors include time-to-result so that patients are not lost to the necessary treatment, and cost, which must be low enough to be incorporated into existing control programs [7,55].

A major challenge in schistosomiasis diagnosis is the lack of true gold-standard test, *i*.*e*., a test with 100% specificity and 100% sensitivity. It is well known that the standard KK test may have a sensitivity as low as 25% in the low transmission setting [64]. An ideal situation would be to have different “gold standards”. For a more accurate sensitivity assessment, the “gold standard” would be repeated KK slides. In this context, considerable effort has been made to develop alternative diagnostic tools. As highlighted in the introduction, the CCA-ICT has been heavily scrutinized in Africa as an alternative to the KK test. In the present investigation, we have studied the performance of CCA-ICT in non-endemic and endemic areas for schistosomiasis mansoni located in two municipalities in Minas Gerais state, Brazil. The CCA-ICT requires visual interpretation and scoring, and when the color reaction is scored as ‘trace’ determining a true cutoff for a negative diagnosis is ambiguous, as noted in previous African studies [56–59]. The issue is important, as sensitivity and specificity are influenced by the interpretation of ‘trace’ as either a positive or negative diagnosis. In order to resolve the matter, we employed ROC curve analysis to understand how the adjudication of the cutoff impacts sensitivity and specificity. We found that when a trace score was considered as a positive diagnosis of infection, the CCA-ICT sensitivity increased resulting in fewer false negative cases. Although this interpretation increases the number of false positives, it is still preferable to missing the diagnosis of infected individuals, which can sustain both transmission of the disease and morbidity. Trace positive reactions may indicate infected individuals who are not (yet) passing eggs in stool, low intensity infections, re-infection after treatment [60] or incomplete clearance of the parasite by PZQ. Although the capture of false positives for treatment isn’t a concern given PZQ’s excellent safety profile, the use of an imperfect diagnostic criterion may incur higher costs as a result of administering unwarranted treatment [58–59,61–62].

In Africa, the latest data for the CCA-ICT suggest that it performs at least as well as the KK test, applied in the WHO standard format or variations thereof [39,57,63–65]. As the drug delivery strategy most often employed in sub-Saharan Africa is preventative chemotherapy (PCT), whereby PZQ treatment is offered at the community level without recourse to individual diagnosis, the recommendations are that the CCA-ICT could replace the KK test. In Brazil, by contrast, individual diagnosis is recommended by the Brazilian Ministry of Health prior to administration of drug therapy [66–67] and here the concern for the CCA-ICT is the number of missed diagnoses (false negatives), among those who are lightly or moderately infected, either when the trace CCA-ICT score is considered as negative (31% false negatives) or positive (14.6% false negatives) based on the particular ROC curve analysis implemented. Our data for false negative rates are consistent with those reported for the CCA-ICT among those with light and moderate infection intensities in Africa, *e*.*g*., approximately 26% in pre-school-age children [64], and 12–18% [63], 23% [68] and 10–44% in school-age children [69] (See S1 Table for a data synopsis of CCA-ICT performances in previous studies).

Our data are also consistent with reports demonstrating that, like the KK test, the CCA-ICT is less reliable in detecting low intensities of infection [39,59,61,65,70]. We found that, among the seven “2KK POS endemic area” adjudicated as negative by the CCA-ICT, five had low and two had moderate intensities of infection. For those with light infection intensities, fluctuations between positive and negative status for both the 2KK and CCA-ICT were noted with the conclusion that more than one urine or stool sample should be collected on different days to increase the KK and CCA-ICT agreement [71]. Also, it is possible that genetic variability in *S*. *mansoni* and its impact on CCA may contribute to the differences in the performance of CCA-ICT [57,72]. Overall, the significant chance of missed diagnoses by the CCA-ICT would lessen its attractiveness as standalone tool for individual diagnosis: the KK test, in some variation, would still have to be employed or perhaps a more rigorous combination of different tests. This in turn adds effort and cost to any diagnostic intervention. In spite of the unreliability of the CCA-ICT to detect light infections, there was a significant positive correlation between the CCA-ICT scores and the 2KK test by both the Spearman rank test (r = 0.625) and the Kappa test (κ = 0.663). Certainly, for heavy infections intensities at or over 400 epg, all of the CCA-ICT scores were either strong (+++) or medium (++). This suggests that the CCA-ICT has little difficulty in identifying those who are heavily infected. The association identified in the current Brazilian setting between the strength of the CCA-ICT scores and infection intensity as judged by the 2KK test has been noted in Africa [59,61,63–65,68–69,73–74] with the suggestion that the CCA-ICT is a viable alternative to the 2KK test in areas of moderate to high prevalence of *S*. *mansoni* infection.

Of interest is our finding of positive CCA-ICT scores among nine of the 41 subjects in the “2KK-NEG non-endemic area” group. In spite of our best efforts to ensure that we were working with a non-endemic group, it is nonetheless possible that these individuals harbored light infections or perhaps single-sex or pre-patent infections. Absenting these considerations, several hypotheses have been put forward to explain the false positivity of the CCA-ICT. According to Van Dam et al. [75–76], there is the possibility of non-specific cross-reactivity with Lewis-X tri-saccharide epitopes in inflammatory biomarkers present in circulating granulocytes. Lewis-X determinants have also been identified in nematode parasites [77]. On this particular point, our data could not offer an explanation as only nine of the 130 subjects were infected with other helminths. We did ascertain, however, that the accuracy of the CCA-ICT was not influenced by the presence or absence of intestinal protozoa.

Using the 2KK test as the reference, the sensitivity (85.4%), NPV (82.1%), specificity (78%) and PPV (82%) of the CCA-ICT (Fig 4C) were comparable to the results reported in the literature, *e*.*g*., a range of 81.4 to 91% for sensitivity, 84.0 to 95.5% for NPV, 47 to 81.0% for specificity and 36 to 84.0% for PPV [58–59,61,70] (S1 Table). Similar data for sensitivity and specificity were also recorded when comparing data from multiple-CCA-ICT and stool samples or a combination of several diagnostic methods were used in order to determine the “real” status of infection [39,62–63,65,74,80] (S1 Table).The efficacy of the CCA-ICT was also compared with the performance of the SEA-ELISA and SWAP-ELISA which measure antibody and are considered the most sensitive techniques to diagnose exposure to schistosomiasis [43,78–79]. However, the major drawback of antibody-based assays is their inability to differentiate between past and current infections which make these tests inadequate to assess disease prevalence in populations that have received treatment [38]. Our ROC curve analyses showed that the discriminative power of the CCA-ICT (AUC = 0.817) was greater than either the SEA-ELISA (AUC = 0.744) or the SWAP-ELISA (AUC = 0.704). Even when combining the results of the 2KK, SEA and SWAP assays (AUC = 0.795), the CCA-ICT maintained a better performance. In this regard, the advantages of the quick and easy-to-use CCA-ICT for POC diagnosis over expertise-driven and lab-based tests are abundantly clear.

The exceptions to the Brazilian Ministry of Health’s recommendation of individual diagnosis prior to treatment are in areas where prevalence rates are between 15 and 25% (household co-inhabitants may receive treatment without diagnosis) and above 25% whereby community therapy may be administered to school children and preschoolers without prior diagnosis [66]. In these settings, the CCA-ICT could provide added value given its ease of use, robustness and assuming that the additional cost can be accommodated into the health system infrastructure. However, the long-standing Brazilian Ministry of Health’s strategy to drive prevalences below 25% [66,67,81] is proving successful [66,82–84] such that individual diagnoses will be increasingly required. In these settings the value of the CCA-ICT as the sole diagnostic test is likely to be less.

## Supporting Information

S1 Table Comparative performance of the CCA-ICT. (PDF)
