# Supplementary material for: Evaluation of the CCA Immuno-Chromatographic Test to Diagnose Schistosoma mansoni in Minas Gerais State, Brazil
Source: PLoS Negl Trop Dis. 2016 Jan 11;10(1):e0004357. doi: 10.1371/journal.pntd.0004357 (PMC4709075; doi:10.1371/journal.pntd.0004357)
Supplement: S1 Table — (PDF) [file pntd.0004357.s001.pdf]

Supplementary Table 1. Comparative performance of the CCA-ICT

	"n"	Reference test[#]	CCA trace	Sen (%)	Spec (%)	PPV (%)	NPV (%)	AUC	Prev % (n)	FP % (n)	FN % (n)	kappa	Healthy Control (n)
**Silveira et al, 2015**	130	2KK[#]	Neg	68.7	97.6	97.1	72.7	0.832	-	2.4 (1)	31.3 (15)	0.663	41
	130		Pos	85.4	78.0	82.0	82.1	0.817	-	22 (9)	14.6 (7)	0.663	41
	56	2KK/SEA/SWAP-ELISA[1]	Pos	86.8	72.2	-	-	0.795	-	27.8 (5)	13.2 (5)	-	-
Van Dam et al., 2004	49	2KK	Pos	100.0	87.0	79.6[2]	0[2]	-	-	-	-	-	45
Stothard et al., 2006	590	2KK	Pos	83.0	81.0	84.0	84.0	-	52	-	-	-	-
Legesse et al., 2007	251	2KK/MIF[3]		82.1	75.9[4]	77.8	48.4	-	60.2	58.3 (42)	17.9 (32)	-	58
Legesse et el., 2008	184	2KK/MIF		76.9	43.4	50.0	71.9	-	36.4	56.6 (60)	23.1 (18)	-	-
Standley et al., 2010a	683	2KK	Neg	63.5	86.4	69.1	83.2	-	34.6	13.6 (56)	-	-	-
			Pos	78.2	66.3	52.6	86.4	-	34.6	37.7 (139)	-	-	-
Standley et al., 2010b	171	2KK	Neg	87.7	68.1	86.1	71.1	-	68.6	-	29 (34)	-	-
			Pos	99.1	19.1	73.4	90.0	-	68.6	28.3 (15)	5.8 (7)	-	-
Shane et al., 2011	482	6KK	Neg	94.2	59.4	-	-	-	38.8	-	-	-	-
	413	2KK/2CCA/SWAP-ELISA[5]	Neg	96.3	74,7	-	-	-	-	-	-	-	-
Coulibaly et al., 2011	A Low[6] (146)	9KK[9]	Neg	56.3	93.9	81.8	81.4	-	32.9	6.1 (6)	43.8 (21)	0.54	-
	B moderate[7] (130)		Neg	69.6	91.8	90.6	72.7	-	53.1	8.2 (5)	30.4 (21)	0.60	-
	C co-endemic[8] (170)		Neg	89.6	84.6	98.6	40.7	-	91.8	15.4 (2)	10.4 (16)	0.49	-
Stothard et al., 2011	242	4KK/percoll/FLOTAC[10]	Neg	59.3	95.6	81.4	87.9	-	-	-	-	-	-
			Pos	81.4	69.9	46.6	92.1	-	-	-	-	-	-
Ashton et al., 2011	276	2KK	Neg	65.2	95.8	83.3	89.4	-	-	-	-	-	-
			Pos	89.1	74.2	52.9	95.5	-	-	-	-	-	-
Tchuem Tchuenté et al., 2012	A Low[11] (138)	9KK[9]	Pos	82.0	64.7	56.9	86.4	-	36.2	35.2 (31)	18 (9)	0.42	-
	B moderate[12] (245)		Pos	82.4	62.3	84.8	58.1	-	71.8	37.7 (26)	17.6 (31)	0.43	-
	C co-endemic[13] (242)		Pos	87.7	42.5	78.6	72.5	-	64	42.5 (37)	12.3 (19)	0.47	-
	All (625)		Pos	84.5	61.5	77.4	71.8	0.72	61	38.5 (94)	15.5 (59)	0.47	-
Navaratnam et al., 2012	569	2KK	Neg	64.4	71.8	51.5	81.3	-	32.2(183)	48.5 (111)	18.5 (34)	-	-
			Pos	81.4	53.2	44.7	86.0	-	32.2(183)	-	-	-	-

Souza Figueiredo et al., 2013	Low[14] (333)	SEA-ELISA	Pos	71.8	100	-	-	0.570	7.2	-	21.7	-	-
	Moderate[15] (337)		Pos	66.5	100	-	-	0.631	16.9	-	2	-	-
	High[16] (255)		Pos	62.7	100	-	-	0.635	38.8	-	2	-	-
	All (925)		Pos	66.9	100	-	-	-	-	-	26.1	-	-
Dawson et al., 2013	< 3[17] (42)	4KK	Pos	69.2	62.1	45.0	81.8	-	31	-	-	-	-
	3-5[18] (40)		Pos	95.8	50.0	74.2	88.9	-	60	-	-	-	-
Coulibaly et al., 2013	242	4KK/2CCA (trace neg)[19]	Neg	69.7	100.0	-	77.4	-	23.1	-	-	0,47	-
			Pos	89.1	59.3	-	84.9	-	-	-	-	0,23	-
Erko et al.. 2013	620	6KK	Neg	93.0	64.6	-	89.1	0.797	53.1	35.4 (103)	7 (23)	0.59	100
		6KK/3CCA[20]	Neg	89.9	100	-	78.2	0.926	-	0	10.1 (46)	-	-
Colley et al.. 2013	Cameron (733)	2KK/CCA/PCR[21]	Pos	87.0	74.0	-	-	-	38.4	47.4 (208)	9.8 (27)	-	-
	Côte d'Ivoire (607)		Pos	78.0	92.0	-	-	-	47.9	14.4 (42)	14.2 (38)	-	-
	Ethiopia (620)		Pos	92.0	94.0	-	-	-	43.0	44.8 (158)	6 (16)	-	-
	Kenya (1,845)		Pos	86.0	70.0	-	-	-	15.1	44.4 (664)	13.2 (35)	-	-
	Uganda (500)		Pos	89.0	56.0	-	-	-	25	53.1 (199)	8.8 (11)	-	-
	All countries (4,305)		Pos	86.0	72.0	-	-	-	28.9	43 (1,271)	10.6 (127)	-	-
Adriko et al., 2014	A Low (100)	2KK	Pos	75	54	13	96	-	8	-	-	0.1	-
		6KK		75	55	19	94	-	13	-	-	0.1	-
	B moderate (200)	2KK	Pos	87	47	32	92	-	23	-	-	0.2	-
		6KK		84	52	46	87	-	33	-	-	0.3	-
	C moderate (200)	2KK	Pos	96	41	48	95	-	36	-	-	0.3	-
		6KK		92	50	69	84	-	55	-	-	0.4	-
	All (500)	2KK	Pos	91	47	36	94.1	-	25	-	-	0.3	-
	All (469)	6KK		88	52	53	88	0.70	38	-	12 (21)	0.4	-

CCA-ICT: Circulating Cathodic Antigen-Immuno-Chromatographic; "n": number of individuals; [#]Reference test to calculate the performance to CCA; Sen: sensitivity; Spec: Specificity; PPV: Positive Predictive Value; NPV: Negative Predictive Value; AUC: Area under the curve; Prev: Prevalence calculated by fecal microcopy (Kato-Katz); FP: Number of false positives; FN: Number of false negatives; [#]2KK= Kato-Katz test (2 slides per stool sample); [1]Combined results of 2KK + ELISA (enzyme-linked immunosorbent assay); SEA: soluble egg antigen; SWAP: soluble (adult) worm antigen preparation; [2]Value from Legesse et al., 2007; [3]Combined results of 2KK and formol-ether concentration (MIF) ; [4]Specificity determined using the control subjects; [5] Combined results of 2KK+2CCA+SWAP-ELISA by LCA (Latent class analysis); [6]Low endemicity (10-24% prevalence); [7]Moderate transmission (25-49% prevalence); [8]co-endemic for *S. mansoni* and *S. haematobium*; [9]9KK: Kato-Katz test (3 slides per stool sample); [10]Combined results of 4KK (2 slides per stool sample) and percoll and FLOTAC; [11]Setting A: low endemicity of single *S. mansoni* transmission; [12]Setting B: moderate endemicity of single *S. mansoni* transmission; [13]Setting C: Mixed infections of *S. mansoni* and *S. haematobium*; [14]Low transmission (< 10% prevalence); [15]Moderate transmission (10-25% prevalence); [16]High transmission (>25% prevalence); [17]Children aged under 3 years; [18]Children between 3-5 years; [19]Combined results of 4KK+2CCA (considering trace results as negative); [20]Combined results of 6 Kato-Katz (2 slides per stool sample) and triple urine CCA cassette; [21]Combined results of Kato-Katz test (2 slides per stool sample), one CCA test and *Schistosoma*-specific PCR (polymerase chain reaction) by LCA (Latent class analysis);

References for Supplementary Table 1

van Dam GJ, Wichers JH, Ferreira TM, Ghati D, van Amerongen A, Deelder AM: Diagnosis of schistosomiasis by reagent strip test for detection of circulating cathodic antigen. J Clin Microbiol 2004, 42(12):5458-61.

Stothard JR, Kabatereine NB, Tukahebwa EM et al: Use of circulating cathodic antigen (CCA) dipsticks for detection of intestinal and urinary schistosomiasis. Acta Trop 2006, 97:219–28.

Legesse M, Erko B. Field-based evaluation of a reagent strip test for diagnosis of Schistosoma mansoni by detecting circulating cathodic antigen in urine before and after chemotherapy. Trans R Soc Trop Med Hyg. 2007, 101(7):668-73.

Legesse M & Erko B. Field-based evaluation of a reagent strip test for diagnosis of schistosomiasis mansoni by detecting circulating cathodic antigen (CCA) in urine in low endemic area in Ethiopia. Parasite. 2008. 15. 151-155

Standley CJ, Adriko M, Arinaitwe M, Atuhaire A, Kazibwe F, Fenwick A, Kabatereine NB, Stothard JR: Epidemiology and control of intestinal schistosomiasis on the Sesse Islands, Uganda: integrating malacology and parasitology to tailor local treatment recommendations Parasites & Vectors 2010a, 3:64.

Standley CJ. Lwambo NJS. Lange CN. Kariuki HC. Adriko M. Stothard JR. Performance of circulating cathodic antigen (CCA) urine-dipsticks for rapid detection of intestinal schistosomiasis in schoolchildren from shoreline communities of Lake Victoria. Parasites & Vectors 2010b. 3:7

Shane HL, Verani JR, Abudho B, Montgomery SP, Blackstock AJ, et al: Evaluation of Urine CCA Assays for Detection of Schistosoma mansoni Infection in Western Kenya. PLoS Negl Trop Dis 2011, 5(1): e951. doi:10.1371/journal.pntd.0000951

Coulibaly JT, Knopp S, N'Guessan NA, Silue KD, Furst T, et al.: Accuracy of urine circulating cathodic antigen (CCA) test for Schistosoma mansoni diagnosis in different settings of Cote d'Ivoire. PLoS Negl Trop Dis 2011, 5: e1384

Stothard JR, Sousa-Figuereido JC, Betson M, Adriko M, Arinaitwe M, Rowell C, Besiyge F, Kabatereine NB: Schistosoma mansoni Infections in Young Children: When Are Schistosome Antigens in Urine, Eggs in Stool and Antibodies to Eggs First Detectable? PLoS Negl Trop Dis 2011, 5(1): e938. doi:10.1371/journal.pntd.0000938

Ashton RA, Stewart BT, Petty N, Lado M, Finn T, Brooker S, Kolaczinski JH Trop: Accuracy of circulating cathodic antigen tests for rapid mapping of Schistosoma mansoni and S. haematobium infections in Southern Sudan Med Int Health 2011,16(9):1099-103. doi: 10.1111/j.1365-3156.2011.02815.x.

Coulibaly JT, Goran EKN, Utzinger J, Doenhoff MJ and Dawson EM. A new rapid diagnostic test for detection of anti-Schistosoma mansoni and anti-Schistosoma haematobium antibodies. Parasites & Vectors 2013, 6:29.

Tchuem Tchuente LA: Control of soil-transmitted helminths in sub-Saharan Africa: diagnosis, drug efficacy concerns and challenges. Acta Trop 2011, 120 Suppl 1:S4-11.

Navaratnam AM, Mutumba-Nakalembe MJ, Stothard JR, Kabatereine NB, Fenwick A, Sousa-Figueiredo JC: Notes on the use of urine-CCA dipsticks for detection of intestinal schistosomiasis in preschool children. Trans R Soc Trop Med Hyg, 2012 106(10):619-22. doi: 10.1016/j.trstmh.2012.06.010.

Dawson EM., Sousa-Figueiredo JC., Kabatereine NB., Doenhoff MJ. and J. Stothard R.: Intestinal schistosomiasis in pre school-aged children of Lake Albert, Uganda: diagnostic accuracy of a rapid test for detection of anti-schistosome antibodies. Trans R Soc Trop Med Hyg 2013,107: 639–647.

Sousa-Figueiredo JC, Betson M, Kabatereine NB, Stothard JR: The Urine Circulating Cathodic Antigen (CCA) Dipstick: A Valid Substitute for Microscopy for Mapping and Point-Of-Care Diagnosis of Intestinal Schistosomiasis. PLoS Negl Trop Dis 2013, 7(1): e2008. doi:10.1371/journal.pntd.0002008

Coulibaly JT, Goran EKN, Utzinger J, Doenhoff MJ and Dawson EM. A new rapid diagnostic test for detection of anti-Schistosoma mansoni and anti-Schistosoma haematobium antibodies. Parasites & Vectors 2013, 6:29.

Erko B, Medhin G, Teklehaymanot T, Degarege A, Legesse M: Evaluation of urine-circulating cathodic antigen (Urine-CCA) cassette test for the detection of Schistosoma mansoni infection in areas of moderate prevalence in Ethiopia. Trop Med Int Health 2013,18: 1029–1035

Colley DG, Binder S, Campbell C, King CH, Tchuem Tchuente LA, N'Goran EK, Erko B, Karanja DM, Kabatereine NB, van Lieshout L et al: A five-country evaluation of a point-of-care circulating cathodic antigen urine assay for the prevalence of Schistosoma mansoni. Am J Trop Med Hyg 2013, 88(3):426-432.

Adriko M, Standley CJ, Tinkitina B, Tukahebwa EM, Fenwick A, Fleming FM, Sousa-Figueiredo JC, Stothard JR, Kabatereine NB: Evaluation of circulating cathodic antigen (CCA) urine-cassette assay as a survey tool for Schistosoma mansoni in different transmission settings within Bugiri District, Uganda. Acta Trop 2014, 136:50-7. doi: 10.1016/j.actatropica.
